# Usual physical activity and subsequent hospital usage over 20 years in a general population: the EPIC-Norfolk cohort

**DOI:** 10.1186/s12877-020-01573-0

**Published:** 2020-05-06

**Authors:** Robert Luben, Shabina Hayat, Nicholas Wareham, Paul Pharoah, Kay-Tee Khaw

**Affiliations:** 1 Department of Public Health and Primary Care, Institute of Public Health, University of Cambridge, Cambridge, UK; 2 MRC Epidemiology Unit, University of Cambridge School of Clinical Medicine, Cambridge, UK

## Abstract

**Background:**

While physical activity interventions have been reported to reduce hospital stays, it is not clear if, in the general population, usual physical activity patterns may be associated with subsequent hospital use independently of other lifestyle factors.

**Objective:**

We examined the relationship between reported usual physical activity and subsequent admissions to hospital and time spent in hospital for 11,228 men and 13,786 women aged 40–79 years in the general population.

**Methods:**

Participants from a British prospective population-based cohort study were followed for 20 years (1999–2019) using record linkage to document hospital usage. Total physical activity was estimated by combining workplace and leisure time activity reported in a baseline lifestyle questionnaire and repeated in a subset at a second time point approximately 12 years later.

**Results:**

Compared to those reporting no physical activity, participants who were the most active had a lower likelihood of spending more than 20 days in hospital odds ratio (OR) 0.88 (95% confidence interval (CI) 0.81–0.96) over the next 20 years after multivariable adjustment for age, sex, smoking status, education, social class and body mass index. Participants reporting any activity had a mean of 0.42 fewer hospital days per year between 1999 and 2009 compared to inactive participants, an estimated potential saving to the National Health Service (NHS) of £247 per person per year, or approximately 7% of UK health expenditure. Participants who remained physically active or became active 12 years later had lower risk of subsequent hospital usage than those who remained inactive or became inactive, p-trend < 0.001.

**Conclusion:**

Usual physical activity in this middle-aged and older population predicts lower future hospitalisations - time spent in hospital and number of admissions independently of behavioural and sociodemographic factors. Small feasible differences in usual physical activity in the general population may potentially have a substantial impact on hospital usage and costs.

## What is already known on this subject

- Pre-admission physical activity interventions have been shown to lower hospital length of stay.
- Usual physical activity is associated with lower rates of mortality from all causes, cardiovascular disease and many non-fatal diseases in the general population, but few studies have examined usual physical activity as a predictor of hospital usage.

## What this study adds

- Usual physical activity, assessed using both occupational and leisure-time components validated against heart rate monitoring with individual calibration, predicted lower hospital usage in a British population of men and women followed up over 20 years.
- Modest differences in usual physical activity in the general population may have a potentially substantial impact on future hospital usage and health service costs.

## Introduction

Historically UK government spending on health has risen on average by 3.7% per year since 1948, outpacing economic growth over the period [1, 2]. As a result, health expenditure as a proportion of UK Gross Domestic Product (GDP) has increased from 3.6 to 7.5% over the same period. Approximately a half of government health expenditure is used for hospitals [3]. There are many factors which may influence hospital usage, not all of which are related to ill health while increases in expenditure are only partly explained by demographic changes [4]. Changes in modifiable lifestyle factors have the potential to lower hospital length of stay. There is growing evidence of the effectiveness of preoperative exercise programmes and other pre-admission interventions in reducing hospital length of stay and readmission rates [5–9] but it is unclear whether in the general population, usual physical activity is related to hospital use. Long-term randomised controlled trials (RCTs) of physical activity interventions with health endpoints are not generally feasible, so evidence is largely based on observational studies.

Physical activity is associated with lower rates of mortality from all causes and cardiovascular disease [10–12]. It is also associated with a lower risk of many non-fatal diseases [13–16] but few studies have examined the relationship between usual physical activity in middle and later life and subsequent hospital usage the general population [17]. The measurement of usual physical activity is problematic. Objective measurements, such as accelerometry have only been developed relatively recently and hence studies based on large, free-living, community-based populations with long follow-up have used self-reported activity from questionnaires. Studies with longer follow-up time are less likely to be affected by reverse causality, which is a feature of studies with short duration of follow-up where individuals who report low physical activity at baseline are inactive by virtue of being affected by the outcome of interest. Self-reported physical activity is most often assessed by questions related to leisure-time activities [18, 19]. Few studies capture both occupational and leisure-time activity.

Hospital usage can be measured by total admissions and length of stay over a fixed follow-up period. These non-disease specific outcome measures can be used to examine the overall level of health service usage [20]. Ageing populations put ever-increasing pressure on health care services and it is therefore important to establish if modest differences in modifiable lifestyle behaviours such as physical activity are related to hospitalisation [21–24].

This study examines the relationship between measures of physical activity using a validated physical activity scale, change in physical activity, and subsequent hospital usage, in older men and women living in the general community over a 10-year period, and a subsequent 10-year follow-up period, taking into account a range of demographic and lifestyle factors.

## Materials and methods

The European Prospective Investigation into Cancer in Norfolk (EPIC-Norfolk) is a general population cohort study of men and women aged 40–79 years living in Norfolk recruited from general practices between 1993 and 1997. The response rate for recruitment was approximately 40%. The cohort has similar characteristics to national population surveys except for a lower prevalence of current smokers [25]. The study has ethics committee approval and all participants gave informed, signed consent for study participation including access to medical records. The cohort is flagged for mortality and hospital admissions from linkage to national databases held by NHS Digital and hence there is virtually no loss to follow-up.

At recruitment, participants completed a lifestyle questionnaire where they were asked about their occupational and leisure physical activity. Occupational activity was assessed using a four category question (“sedentary”, “standing”, “moderate physical work” and “heavy manual work”) with examples such as office worker, shop assistant, plumber and construction worker respectively. Leisure activity in both summer and winter was assessed from the number of hours per week spent cycling, attending keep fit classes or aerobics and swimming or jogging. Estimated average hours of leisure activity was calculated as the mean of summer and winter activities and categorised using 0, (0,3.5], (3.5,7] and > 7. A combined score, divided into four ordered categories with individuals labelled as “inactive”, “moderately inactive”, “moderately active” and “active” was created combining leisure and occupational elements. Those who did not complete the activity question were placed in the inactive category. The score was validated against energy expenditure measured by free-living heart rate monitoring with individual calibration [26]. It has been reported to predict all-cause mortality and cardiovascular disease incidence [27].

Participants attending the baseline health examination had their height to the nearest 0.1 kg measured using a stadiometer (Chasemores, UK) and their weight to the nearest 100 g measured in light clothing without shoes (Salter, West Bromwich, UK). Body mass index (BMI) was calculated using measured weight in kilograms divided by the square of measured height in square metres. Two yes/no questions were used to derive smoking status: “Have you ever smoked as much as one cigarette a day for as long as a year?” and, where a positive response was given, “Do you smoke cigarettes now?” Participants also completed questions about their employment and that of their partner with details of both current and past employment recorded. Occupational social class was defined according to the Registrar General’s classification [28, 29]. A list of common UK qualifications was used to establish educational attainment and participants were asked to mark all relevant qualifications. These were then categorised using the highest qualification attained. Participants were asked at baseline “Has the doctor ever told you that you have any of the following?” followed by a list of common conditions including “Heart attack (myocardial infarction)”, “Stroke” and “Cancer”.

Surviving participants were invited to complete a lifestyle questionnaire and attend a health examination (second time-point, “TP2”) between 2006 and 2011 [30] . Questions on physical activity and cigarette smoking, similar to those at baseline, were included in a postal questionnaire, completed by a subset of 9827 of the original cohort. Weight and height were measured on 8094 by clinic staff and body mass index calculated in the same way as at baseline described previously.

### Ascertainment of hospital usage through record linkage

The National Health Service (NHS) in Britain treats residents without charge at the point of service so covers virtually all major health service usage. The EPIC-Norfolk cohort was regularly linked to hospital records from 1999 onwards as previously reported [20]. Briefly, NHS numbers were used to perform linkage to hospital databases between 1999 and 2019. Initially, up to 2009, linkage was made via the East Norfolk Primary Health Care Trust while later, national databases held by NHS Digital were used [31]. All hospital activity for EPIC-Norfolk participants was captured wherever they were treated in England and Wales. Hospital episode statistics (HES) records which included admission and discharge dates were used to calculate time in hospital and numbers of admissions. Contiguous admissions were merged and counted as a single admission.

### Statistical analysis

For the main analysis, 625 men and women who died before 1999 were excluded. Dichotomous variables were created for the socioeconomic status variables. Professional, managerial and technical and non-manual skilled occupations (codes I, II and IIIa respectively) were classed as non-manual while manual skilled, partly skilled and unskilled (codes IIIb, IV and V respectively) were classed as manual. Educational attainment was categorised into “Higher education level” (which includes those with qualifications at secondary level or above) and “Lower education level” (those with no qualifications). The numbers of individuals with missing values for covariables were: 53 BMI, 218 smoking status, 545 social class, 18 education level. Validation of the physical activity measures [26] suggested that participants with missing data be classified inactive.

Logistic regression was used to model hospitalisation outcomes on physical activity category, adjusting for covariables. Several dichotomous outcome categories were calculated based on total admissions and length of stay spanning two periods: 1999–2009 (10-year follow-up) and 1999–2019 (20-year follow-up). Total admissions from 10-year follow-up were used to define “any hospital admissions” and “7 or more admissions” while length of stay from 10-year follow-up was used to create “greater than 20 hospital days”. These thresholds were chosen to represent those with higher levels of hospital usage and were consistent with previous work [20]. Dichotomous outcome categories based on 20-year follow-up and having approximately the same proportion of the population as their 10-year follow-up counterparts include “12 or more admissions” and “greater than 50 hospital days” while “7 or more admissions” and “greater than 20 hospital days” were also calculated for this period to serve as a comparison. Hospital days are defined as the sum of total bed days (overnight stays) and day-cases. Linear regression was used to calculate the absolute difference in adjusted mean bed days between inactive participants and participants reporting any activity.

To address change in physical activity, we also used physical activity measured at TP2 approximately 12 years later as a second baseline. We excluded 105 participants who died prior to 2009, leaving 9722. Multiple imputation was used to address missing values, in particular for body mass index at TP2 where data for 1733 were not available for participants who completed a TP2 questionnaire but did not attend a health examination. Predictive mean matching with 5 multiple imputations and 50 iterations was used with baseline variables BMI, occupational social class and education attainment and TP2 current smoking. Changed-activity categories use combinations of physical activity categories at the baseline and TP2. The category shown as “Inactive/Inactive” is the set of participants who reported being inactive at baseline and remained inactive when asked again at TP2. The group who initially reported any activity but became inactive later is shown as “Any-activity/Inactive” while the other two categories “Inactive/Any-activity” and “Any-activity/Any-activity” were similarly defined.

The cost to the NHS of one bed-day is £496, calculated using the Reference Costs for English Hospitals 2017/18 for elective (5.4 £bn) and non-elective (18 £bn) admissions [32] and the total available beds (approximately 129,200) [33]. The cost per hospital day (overnight stays and day-cases) is £587 when the cost of day-case activity is included (4.4 £bn per year). The reported OECD UK per capita expenditure on health in 2017, was £3375 (exchange rate at the time of writing) [34]. Per-person costs were calculated by multiplying the cost per hospital day and hospital days per person. Percentage of NHS per-capita health expenditure was calculated as the ratio of per-person cost and OECD UK per-capita expenditure.

Adjusted mean hospital days by physical activity category were determined first by calculating hospital days for each one year period restricted to participants surviving to the start of the given year. Linear regression of hospital days on physical activity adjusted for age, sex, occupational social class, educational attainment, current smoking and body mass index was then used. Adjusted means by category were obtained using estimated marginal means. The overall mean difference of days was calculated by taking the mean of the annual differences for each of two periods (1999–2009 and 2009–2019).

Sensitivity analyses were conducted in which the physical activity exposure was dichotomised into inactive and any-activity groups, using the outcome more than 20 hospital days over the period 1999–2019. Multivariable adjusted odds ratios were examined, stratified by sex, age < 65 and ≥ 65 years, manual and non-manual social class, lower (no qualifications) and higher level of education, former or never smoking and current smoking, BMI ≤30, > 30 kg/m^2^, chromic disease (heart attack, stroke or cancer) and no reported chronic disease, survival to the end of follow-up (March 2019) and died during follow-up period. A further multivariable model was performed using the narrower follow-up period of 2004–2019, a minimum of five years after participants reported their level of physical activity excluding participants who died prior to 2004.

All analyses were performed using the R statistical language (R Foundation for Statistical Computing, Vienna, Austria version 3.6.0 with packages ggeffects, knitr, Gmisc, tidyverse, intubate, mice).

## Results

Characteristics of the study population according to the four categories of physical activity score are described in Table 1. Active participants tend to be younger, non-smokers, without chronic disease and have higher educational attainment, however those with manual social class also tend to be more active.

**Table 1:** | Descriptive characteristics by physical activity category measured at baseline 1993–1997

	Total	Inactive (*n* = 7559 30.2%)	Moderately inactive (*n* = 7187 28.7%)	Moderately active (*n* = 5688 22.7%)	Active (*n* = 4580 18.3%)
**Body mass index, kg/m**^**2**^					
Mean ± SD	26.4 ± 3.9	27.0 ± 4.2	26.3 ± 3.9	26.0 ± 3.7	25.9 ± 3.5
**Age, years**					
Mean ± SD	59.0 ± 9.3	62.5 ± 9.1	58.8 ± 9.2	57.1 ± 8.7	56.1 ± 8.4
**Cigarette smoking (n (%))**					
Current	2904 (11.7)	984 (13.2)	770 (10.8)	662 (11.7)	488 (10.7)
Former	10,423 (42.0)	3326 (44.6)	2818 (39.5)	2312 (40.9)	1967 (43.2)
Never	11,469 (46.3)	3151 (42.2)	3540 (49.7)	2678 (47.4)	2100 (46.1)
**Social class dichotomised (n (%))**					
Non-manual	14,717 (60.1)	4394 (60.2)	4791 (67.8)	3261 (58.3)	2271 (50.4)
Manual	9752 (39.9)	2900 (39.8)	2278 (32.2)	2337 (41.7)	2237 (49.6)
**Level of education (n (%))**					
Higher level	15,866 (63.5)	4252 (56.4)	4757 (66.2)	3823 (67.2)	3034 (66.2)
Lower level	9130 (36.5)	3289 (43.6)	2430 (33.8)	1865 (32.8)	1546 (33.8)
**Prevalent disease (n (%))**					
No reported chronic disease	22,721 (91.0)	6606 (87.7)	6573 (91.5)	5246 (92.3)	4296 (93.9)
Self-report chronic disease	2254 (9.0)	927 (12.3)	608 (8.5)	439 (7.7)	280 (6.1)
**Hospital activity 1999–2019**					
No admissions	2483 (9.9)	625 (8.3)	726 (10.1)	613 (10.8)	519 (11.3)
One or more admissions	22,497 (90.1)	6915 (91.7)	6453 (89.9)	5072 (89.2)	4057 (88.7)
**Time in hospital 1999–2019**					
Mean ± SD	34.0 ± 63.7	42.4 ± 68.2	32.9 ± 64.1	29.9 ± 66.4	26.8 ± 48.8
Median (IQR)	14.0 (3.0–41.0)	21.0 (6.0–56.0)	13.0 (3.0–39.0)	11.0 (3.0–33.0)	10.0 (2.8–30.0)
**Number of admissions 1999–2019**					
Mean ± SD	7.8 ± 26.5	8.4 ± 29.0	7.6 ± 24.5	7.8 ± 32.2	6.9 ± 14.8
Median (IQR)	4.0 (2.0–9.0)	5.0 (2.0–9.0)	4.0 (2.0–8.0)	4.0 (2.0–8.0)	4.0 (2.0–8.0)
**Survival to the end of follow-up (n (%))**					
Alive after March 2019	15,919 (63.6)	3732 (49.4)	4746 (66.0)	4047 (71.1)	3394 (74.1)
Died prior to March 2019	9095 (36.4)	3827 (50.6)	2441 (34.0)	1641 (28.9)	1186 (25.9)

Prevalent disease is self-reported heart attack, stroke or cancer at baseline. Higher education level represents those with qualifications to at least secondary level.

In Table 2 odds ratios are shown first age and sex adjusted and then additionally adjusted for social class, educational attainment, BMI and smoking status. For the 10-year follow-up period 1999–2009, outcomes of any hospital admission, 7 or more hospital admissions and more than 20 days stay in hospital are shown according to the baseline physical activity score. The multivariable-adjusted models indicate that participants with a physical activity score of at least moderately inactive had fewer hospital admissions and fewer days in hospital, than those who were inactive. The associations for inactive vs active were OR 0.73 (95% CI 0.65–0.82) p-trend < 0.001 across activity score for seven or more hospital admissions and OR 0.75 (95% CI 0.67–0.83) p-trend < 0.001 for more than 20 hospital days.

**Table 2:** | Multivariable logistic regression of risk factors by physical activity category for hospital admissions and length of hospital stay categories over 10 years (1999 to 2009) and 20 years (1999 to 2019) in 25,014 men and women and 10 years (2009–2019) using the TP2 baseline in 9722 men and women

	Inactive *n* = 7559	Moderately inactive *n* = 7187	Moderately active *n* = 5688	Active n = 4580	p (trend)
**10-year follow-up**					
**Outcome of any hospital admissions (18,179/25014)**					
n (%)	5878 (78%)	5103 (71%)	3980 (70%)	3218 (70%)	
Model 1^a^	1.00	0.87 (0.80–0.94)	0.90 (0.83–0.97)	0.96 (0.88–1.05)	0.373
Model 2^b^	1.00	0.91 (0.84–0.98)	0.91 (0.84–0.99)	0.95 (0.87–1.04)	0.286
**Outcome of seven or more hospital admissions (3462/25014)**					
n (%)	1392 (18%)	891 (12%)	689 (12%)	490 (11%)	
Model 1^a^	1.00	0.76 (0.69–0.83)	0.79 (0.71–0.87)	0.71 (0.63–0.79)	< 0.001
Model 2^b^	1.00	0.80 (0.72–0.88)	0.82 (0.73–0.91)	0.73 (0.65–0.82)	< 0.001
**Outcome of more than 20 hospital days (4976/25014)**					
n (%)	2122 (28%)	1299 (18%)	893 (16%)	662 (14%)	
Model 1^a^	1.00	0.75 (0.69–0.81)	0.72 (0.66–0.79)	0.71 (0.64–0.79)	< 0.001
Model 2^b^	1.00	0.80 (0.74–0.87)	0.77 (0.70–0.84)	0.75 (0.67–0.83)	< 0.001
**20-year follow-up**					
**Outcome of any hospital admissions (22,497/25014)**					
n (%)	6915 (91%)	6453 (90%)	5072 (89%)	4057 (89%)	
Model 1^a^	1.00	1.06 (0.94–1.19)	1.07 (0.95–1.21)	1.08 (0.95–1.22)	0.238
Model 2^b^	1.00	1.11 (0.98–1.24)	1.10 (0.97–1.25)	1.08 (0.95–1.23)	0.274
**Outcome of seven or more hospital admissions (8849/25014)**					
n (%)	2969 (39%)	2490 (35%)	1879 (33%)	1511 (33%)	
Model 1^a^	1.00	0.94 (0.88–1.01)	0.92 (0.85–0.99)	0.94 (0.87–1.02)	0.055
Model 2^b^	1.00	0.98 (0.91–1.05)	0.94 (0.87–1.01)	0.96 (0.89–1.05)	0.194
**Outcome of 12 or more hospital admissions (3989/25014)**					
n (%)	1354 (18%)	1088 (15%)	894 (16%)	653 (14%)	
Model 1^a^	1.00	0.90 (0.82–0.98)	0.95 (0.87–1.05)	0.85 (0.76–0.94)	0.010
Model 2^b^	1.00	0.95 (0.87–1.04)	0.98 (0.89–1.08)	0.87 (0.78–0.97)	0.040
**Outcome of more than 20 hospital days (10,174/25014)**					
n (%)	3800 (50%)	2836 (39%)	1996 (35%)	1542 (34%)	
Model 1^a^	1.00	0.87 (0.81–0.93)	0.82 (0.76–0.88)	0.84 (0.77–0.91)	< 0.001
Model 2^b^	1.00	0.93 (0.86–1.00)	0.86 (0.79–0.93)	0.88 (0.81–0.96)	< 0.001
**Outcome of more than 50 hospital days (5178/25014)**					
n (%)	2065 (27%)	1411 (20%)	994 (17%)	708 (15%)	
Model 1^a^	1.00	0.85 (0.79–0.93)	0.86 (0.78–0.94)	0.81 (0.73–0.89)	< 0.001
Model 2^b^	1.00	0.91 (0.84–0.99)	0.91 (0.83–1.00)	0.84 (0.76–0.94)	0.001
**10-year follow-up from TP2 baseline**					
	Inactive *n* = 3937	Moderately inactive *n* = 2686	Moderately active *n* = 1655	Active *n* = 1444	p (trend)
**Outcome of any hospital admissions (7855/9722)**					
n (%)	3332 (85%)	2127 (79%)	1267 (77%)	1129 (78%)	
Model 1†	1.00	0.93 (0.81–1.06)	0.85 (0.73–0.99)	1.00 (0.85–1.17)	0.484
Model 2‡	1.00	0.97 (0.85–1.11)	0.90 (0.77–1.05)	1.04 (0.88–1.22)	0.922
**Outcome of seven or more hospital admissions (1802/9722)**					
n (%)	874 (22%)	466 (17%)	259 (16%)	203 (14%)	
Model 1^a^	1.00	0.89 (0.78–1.01)	0.81 (0.69–0.94)	0.73 (0.62–0.87)	< 0.001
Model 2^b^	1.00	0.93 (0.82–1.06)	0.84 (0.72–0.99)	0.77 (0.64–0.91)	0.001
**Outcome of more than 20 hospital days (2170/9722)**					
n (%)	1217 (31%)	489 (18%)	273 (16%)	191 (13%)	
Model 1^a^	1.00	0.69 (0.61–0.78)	0.69 (0.59–0.80)	0.57 (0.48–0.68)	< 0.001
Model 2^b^	1.00	0.72 (0.64–0.82)	0.71 (0.61–0.83)	0.60 (0.50–0.72)	< 0.001

^a^ Adjusted for age, sex, manual social class, lower education level, current cigarette smoker, body mass index > 30 kg/m^2^.

Attenuated results were observed for longer follow-up. Odds ratios over the 20-year period 1999–2019 are presented for any hospital admission, ≥7 admissions, ≥12 admissions, > 20 hospital days and > 50 hospital days and associations were OR 0.96 (95% CI 0.89–1.05) p-trend 0.194 for ≥7 admissions, OR 0.87 (95% CI 0.78–0.97) p-trend 0.040 for ≥12 admissions, and OR 0.88 (95% CI 0.81–0.96) p-trend < 0.001 for > 20 hospital days, OR 0.84 (95% CI 0.76–0.94) p-trend 0.001 for > 50 hospital days. Associations for > 20 hospital days and > 50 hospital days were similar, while the inverse association using the threshold of ≥12 admissions was higher than that for the ≥7 admissions threshold.

Physical activity category at TP2 baseline was determined in 9827 men and women. The associations for inactive vs active for 20 hospital days over the subsequent 10-year follow-up period (2009 to 2019) were stronger than those for the first 10-year follow-up period OR 0.60 (95% CI 0.50–0.72) p-trend < 0.001 and similar for 7 or more admissions OR 0.77 (95% CI 0.64–0.91) p-trend 0.001.

Table 3 shows multivariable-adjusted odds ratios for outcome more than 20 hospital days during the 1999–2019 follow-up in participants who were inactive compared to those reporting any activity at baseline, stratified by key variables in subgroups. The directions of the associations did not differ by subgroup. Higher inverse associations were seen in women, in the under 65 s, in those with no chronic disease at baseline and those surviving to the end of follow-up although confidence intervals overlapped in each case. Table 3 also shows that the association for the period 2004–2019, excluding the first 5 years of the outcome period was OR 0.93 (95% CI 0.87–1.00).

**Table 3:** | Multivariable logistic regression of simple physical activity index and more than 20 hospital days in subgroups after 20 years follow-up

	Inactive (n = 7559) (ref)	Any-activity (*n* = 17,455) OR (95% CI)^a^
**Men and women**		
Men (*n* = 11,228)	1	0.92 (0.84–1.01)
Women (*n* = 13,786)	1	0.87 (0.80–0.95)
**By age above and below 65 years**		
Younger than 65 years (*n* = 17,372)	1	0.86 (0.80–0.93)
65 years and older (*n* = 7642)	1	0.91 (0.83–1.01)
**Manual and non-manual social class**		
Non-manual (*n* = 14,717)	1	0.89 (0.82–0.97)
Manual (*n* = 9752)	1	0.89 (0.81–0.99)
**By level of education**		
Higher level (*n* = 15,866)	1	0.91 (0.84–0.98)
Lower level (*n* = 9130)	1	0.87 (0.78–0.95)
**By smoking status**		
Former or never smoker (*n* = 21,892)	1	0.88 (0.83–0.95)
Current smoker (*n* = 2904)	1	0.97 (0.82–1.16)
**By level of body mass index**		
BMI ≤ 30 kg/m^2^ (*n* = 21,158)	1	0.90 (0.84–0.97)
BMI > 30 kg/m^2^ (*n* = 3803)	1	0.86 (0.75–1.00)
**Prevalent disease**		
No reported chronic disease (*n* = 22,721)	1	0.90 (0.84–0.96)
Self-report chronic disease (*n* = 2254)	1	0.94 (0.78–1.14)
**Survival to end of follow-up**		
Alive after March 2019 (*n* = 15,919)	1	0.90 (0.82–0.98)
Died prior to March 2019 (*n* = 9095)	1	0.99 (0.90–1.10)
**Excluding first five years**		
Admissions 2004–2019 (*n* = 23,487)	1	0.93 (0.87–1.00)

^a^ Adjusted for age, sex, manual social class, lower education level, current cigarette smoker, body mass index > 30 kg/m^2^.

Table 4 shows odds ratios by all combinations of change in physical activity category between baseline and TP2 were determined using the TP2 baseline and subsequent 10-year follow-up. The multivariable-adjusted odds ratios comparing “Inactive/Inactive” (the reference) and “Any-activity/Any-activity” were OR 0.66 (95% CI 0.57–0.77) p-trend < 0.001 across changed-activity categories for more than 20 hospital days and OR 0.91 (95% CI 0.78–1.07) p-trend 0.026 for seven or more hospital admissions. Participants who remained physically active or became active had lower risk of subsequent hospital usage than those who remained inactive or became inactive.

**Table 4:** | Multivariable logistic regression of risk factors by change in physical activity category between baseline and TP2 for hospital admissions and length of hospital stay categories over 10 years (2009 to 2019) in 9722 men and women

	Inactive/Inactive *n* = 1441	Any-activity/Inactive *n* = 2496	Inactive/Any-activity *n* = 790	Any-activity/Any-activity *n* = 4995	p (trend)
**Outcome of any hospital admissions (7855/25014)**					
n (%)					
Model 1^a^	1.00	1.16 (0.96–1.39)	1.10 (0.87–1.39)	1.00 (0.85–1.18)	0.246
Model 2^b^	1.00	1.18 (0.98–1.42)	1.15 (0.91–1.47)	1.06 (0.90–1.26)	0.751
**Outcome of seven or more hospital admissions (1802/25014)**					
n (%)					
Model 1^a^	1.00	1.09 (0.93–1.28)	1.00 (0.80–1.25)	0.86 (0.74–1.00)	0.002
Model 2^b^	1.00	1.12 (0.95–1.32)	1.05 (0.83–1.31)	0.91 (0.78–1.07)	0.026
**Outcome of more than 20 hospital days (2170/25014)**					
n (%)					
Model 1^a^	1.00	0.96 (0.83–1.11)	0.78 (0.62–0.96)	0.62 (0.54–0.72)	< 0.001
Model 2^b^	1.00	0.98 (0.84–1.14)	0.81 (0.65–1.01)	0.66 (0.57–0.77)	< 0.001

^a^ Adjusted for age at TP2 and sex. ^b^ Adjusted for age at TP2, sex, baseline manual social class, baseline lower education level, current cigarette smoker at TP2, body mass index > 30 kg/m^2^ at TP2. Multiple imputation was used for 1733 missing BMI at TP2 calculated using baseline BMI and other covariates for participants who completed questionnaires but did not attend a health examination

Supplementary Table S1 shows all terms in a series of multivariable logistic regression models for inactive physical activity (vs any-activity) and various dichotomous outcomes over the period 1999–2019 for all, men and women. Covariables age per 10 years, manual social class, lower education level, current smoking and BMI > 30 kg/m^2^ are modelled; all are independently associated with number of hospital admissions and length of stay. Associations were similar in men and women. The duration outcomes 20 or 50 hospital days were associated with the binary physical activity classification although associations with numbers of hospital admissions were attenuated.

Supplementary Table S2 shows the adjusted mean hospital days for inactive and any-activity participants by year, and the absolute difference in days between the categories. The mean of the differences was calculated for 1999–2009 using baseline physical activity and 2009–2019 using physical activity at TP2 and cumulative costs were determined assuming £587 per hospital day.

The difference in multivariable adjusted mean hospital days between inactive participants and participants reporting any activity was 0.42 days per year over the first 10 years of follow-up, an estimated potential saving to the NHS of £247 per person per year or approximately 7% of health expenditure. The difference in hospital days over the subsequent 10 years (2009–2019) was slightly higher, with any-activity participants having 0.46 fewer hospital days, an estimated potential saving of £268 or approximately 8% of health expenditure.

## Discussion

Usual physical activity assessed at baseline survey in 1993–1997 was inversely associated with future hospital usage independently of sociodemographic and lifestyle factors in this middle-aged and older cohort of men and women over a 20-year follow-up period. Compared to study participants who were inactive, active participants had a lower likelihood of having more than 20 hospital days or more than 12 admissions. Stronger associations were seen over a 10-year follow-up period with moderate inactivity or greater being associated with lower risk of seven or more hospital admissions or more than 20 hospital days. There was a dose response over physical activity categories over both the 10-year and 20-year follow-up periods for both hospital duration and number of admissions. There are a number of possible explanations for these findings.

### Strengths and limitations of study

Reverse causality may partly explain the associations we observed. Participants may be physically inactive occupationally or less able to take part in leisure time activity because of known or preclinical illness which may also predispose to increased later hospitalisation [35]. However, sensitivity analyses excluding those with a self-reported chronic disease at baseline (heart attack, stroke or cancer), who might have lower physical activity, did not differ materially from the main findings. Also, a sensitivity analysis excluding hospital admissions occurring in the first 5 years of follow-up (the period 2004–2019), that is, those who were more likely to have preclinical illness and lowered physical activity, again did not show materially different associations.

Confounding is a major issue in examining the relationship between lifestyle factors and health outcomes. Individuals who are more physically active are likely to differ from those who are less active with respect to other factors relating to the likelihood of future hospitalisation including age, sex, smoking, body mass index, social class and education. However, the associations were consistent after multivariable-adjustment for these factors and after stratification by these potential confounding variables.

As we examined total hospital usage over long time periods, individuals who died during the follow-up period did not use hospital services for the full period. This may have affected the results if there was differential mortality by physical activity, whereby study participants who were inactive were more likely to have died earlier than the more active participants and hence less likely to use hospital services for the full follow-up period. Sensitivity analysis models restricted to those surviving to the end of 20-year follow-up showed stronger associations of physical activity with lower hospital use than models using the whole population, including those who died during the follow-up period, suggesting there was some attenuation due to selective follow-up.

This study has several strengths. Few studies have examined the physical activity of middle-aged and older men and women and their subsequent healthcare utilisation. The literature falls into two groups, studies based on exercise interventions and observational studies. While most intervention studies provide some evidence that a physically active lifestyle improves health, intervention protocols vary and differences in dropout rates between groups in RCTs limit generalisability [36]. Intervention studies may also typically have smaller study size and shorter follow-up time and while observational studies are generally larger, there are few studies comparable in size to the present study. Our study, being well characterised, allowed adjustment for a broad range of relevant factors. We also used linked hospital data and did not depend on self-reported outcome data. Many studies are based on particular population groups or particular disease outcomes and some rely on self-selection to exercise programs. Few studies examine free living community-based populations [37, 38], however we used a prospective cohort design and were able to examine hospital usage over a long follow-up period with a reliable population-based denominator.

Our study was based on a free-living population of older men and women living in the general community in the United Kingdom where the NHS provides health care free at the point of delivery. Potential major confounders such as income, and ability to pay that might therefore affect and limit access and use of health services, are less likely to apply in this study. The NHS also enables record linkage for virtually complete follow-up of the population. Though admissions to private hospitals in Norfolk were not included in our data which only counts NHS hospitals, the use of private hospitals in Norfolk was minimal in comparison with the use of NHS facilities.

Measurement of usual occupational and leisure time physical activity was assessed using a self-reported questionnaire. Objective measures such as accelerometry and similar techniques were not available when the EPIC-Norfolk cohort was recruited. However, the physical activity score used was previously validated using heart rate monitoring with individual calibration and based on both occupational and leisure-based components of physical activity.

It is also clear that a single measurement of physical activity is insufficient to determine accurately usual levels of activity over the life course. Events such as retirement or illness or progressive ageing related conditions such as frailty may result in a change to the amount of physical activity undertaken [39]. While we are unable to establish the length of time over which consistent physical activity was maintained, we were able to examine longitudinal measurements of physical activity at two time-points in a subset of participants. The associations observed at the later time-point were comparable with (in fact stronger than) those observed at the first time-point, despite the cohort mean age being approximately 10 years older and having a much higher proportion of retirees. Change in behaviour over the 20-year follow-up period is a more likely explanation for the attenuated associations observed, rather than age or employment status. Participants who remained inactive or became inactive had the highest risk of subsequent hospitalisation. Additionally, random measurement error is likely only to attenuate associations, and therefore unlikely to explain any of the associations observed between physical activity and hospitalisation.

### Comparison with other studies

Physical activity has been associated with many health benefits including protection against cardiovascular [10, 12, 40] and many other chronic diseases [41, 42] so there are many plausible reasons why it might also be associated with lower hospitalisations from individual conditions. Chronic conditions such as cardiovascular disease remain leading causes of hospitalisation. However, in this study, we were able to examine total hospital usage in a general population irrespective of cause of admission.

Small increases in physical activity have been reported to obtain cost savings for health services by reducing hospital admissions [17, 22, 43–45] with many studies reporting reductions of length of stay after preoperative physical activity interventions. Our study has observed a 12–13% lower risk of long stay and high numbers of admissions by physical activity category. The mean difference in bed days between inactive and any-activity participants in our study was 0.42 days per year over the first 10 years of follow-up. Assuming a cost of £587 per hospital day (inpatient bed-days and day-cases), the potential saving to the NHS is approximately £247 per person per year for every inactive person who starts to undertake at least some exercise, or about 7% of UK per capita health expenditure. Similar results were observed 10 years later when participants were aged 50–90 years. Calculations such as these are unavoidably crude but serve to illustrate the significant financial contribution, when scaled nationally, that modest changes in lifestyle can achieve quite apart from the obvious personal gain from the reduction in risk of being hospitalised.

While there is evidence suggesting that pre-admission physical activity programmes may lower duration of hospital stay [5–8, 46], these are short term, requiring resources and targeted at only a limited number of individuals. Our data indicate that usual physical activity patterns in the general population predict hospital usage over the subsequent 2 decades.

## Conclusions and policy implications

Usual physical activity in this middle-aged and older population predicts lower future hospitalisations - time spent in hospital and number of admissions independently of behavioural and sociodemographic factors. Small, feasible differences in usual physical activity in the general population may potentially have a substantial impact on hospital usage and costs.

## Supplementary information

**Additional file 1: Table S1.** Multivariable logistic regression of risk factors for any hospital admissions, ≥7 hospital admissions and > 20 days of hospital stay from 1999 to 2019 in 25,014 men and women. **Table S2.** Adjusted mean hospital days by physical activity category for two periods, mean difference in days and cumulative cost, 1999–2009 using baseline physical activity and 2009–2019 using physical activity at TP2. **Figure S1.** Flow diagram showing numbers of participants at various stages in the EPIC-Norfolk study including invitations, consents, attendance at health examinations and questionnaire completion.

## Data Availability

The authors will make the dataset available under a Data Transfer Agreement to any bona fide researcher who wishes to obtain the dataset in order to undertake a replication analysis. Although the dataset is anonymised, the breadth of the data included and the multiplicity of variables that are included in this analysis file as primary variables or confounding factors, means that provision of the dataset to other researchers without a Data Transfer Agreement would constitute a risk. Requests for data sharing/access should be submitted to the EPIC Management Committee (epic-norfolk@mrc-epid.cam.ac.uk).

## Abbreviations

BMI: Body mass index
CI: Confidence interval
EPIC-Norfolk: European Prospective Investigation into Cancer in Norfolk
GDP: Gross Domestic Product
HES: Hospital episode statistics
OECD: Organisation for Economic Co-operation and Development
OR: Odds ratio
TP2: Time-point two

## References

[1] Stoye G (2018). Does the NHS need more money and how could we pay for it? The Health Foundation, the Institute for Fiscal Studies, The King’s Fund and the Nuffield Trust.

[2] Lloyd T (2015). The Health Foundation. Funding Overview Historical Trends in The UK.

[3] Office for National Statistics (2017). Healthcare expenditure, UK Health Accounts.

[4] Appleby J (2013). Spending on health and social care over the next 50 years: why think long term?.

[5] Weinstein H, Bates AT, Spaltro BE, Thaler HT, Steingart RM (2007). Influence of preoperative exercise capacity on length of stay after thoracic Cancer surgery. Ann Thorac Surg.

[6] Pouwels S, Stokmans RA, Willigendael EM, Nienhuijs SW, Rosman C, van Ramshorst B (2014). Preoperative exercise therapy for elective major abdominal surgery: a systematic review. Int J Surg.

[7] Perry R, Scott LJ, Richards A, Haase AM, Savović J, Ness AR, et al. Pre-admission interventions to improve outcome after elective surgery—protocol for a systematic review. Syst Rev. 2016;5. 10.1186/s13643-016-0266-9. 10.1186/s13643-016-0266-9 PMC4878054 27216584

[8] Steffens D, Beckenkamp PR, Hancock M, Solomon M, Young J (2018). Preoperative exercise halves the postoperative complication rate in patients with lung cancer: a systematic review of the effect of exercise on complications, length of stay and quality of life in patients with cancer. Br J Sports Med.

[9] de Souto Barreto P, Rolland Y, Vellas B, Maltais M (2019). Association of Long-term Exercise Training With Risk of Falls, Fractures, Hospitalizations, and Mortality in Older Adults: A Systematic Review and Meta-analysis. JAMA Intern Med.

[10] Nocon M, Hiemann T, Müller-Riemenschneider F, Thalau F, Roll S, Willich SN (2008). Association of physical activity with all-cause and cardiovascular mortality: a systematic review and meta-analysis. Eur J Cardiovasc Prev Rehabil.

[11] Cooper R, Kuh D, Hardy R, Group MR (2010). Objectively measured physical capability levels and mortality: systematic review and meta-analysis. BMJ..

[12] Bennett DA, Du H, Clarke R, Guo Y, Yang L, Bian Z (2017). Association of Physical Activity with Risk of major cardiovascular diseases in Chinese men and women. JAMA Cardiol.

[13] Reiner M, Niermann C, Jekauc D, Woll A (2013). Long-term health benefits of physical activity – a systematic review of longitudinal studies. BMC Public Health.

[14] Warburton DE, Charlesworth S, Ivey A, Nettlefold L, Bredin SS (2010). A systematic review of the evidence for Canada’s physical activity guidelines for adults. Int J Behav Nutr Phys Act.

[15] Knight JA (2012). Physical inactivity: associated diseases and disorders. Ann Clin Lab Sci.

[16] Mok A, Khaw K-T, Luben R, Wareham N, Brage S (2019). Physical activity trajectories and mortality: population based cohort study. BMJ..

[17] Peeters GMEE (Geeske), Gardiner PA, Dobson AJ, Brown WJ. Associations between physical activity, medical costs and hospitalisations in older Australian women: Results from the Australian Longitudinal Study on Women’s Health. J Sci Med Sport. 2018;21:604–608. 10.1016/j.jsams.2017.10.022 29102460

[18] Bonnefoy M, Normand S, Pachiaudi C, Lacour JR, Laville M, Kostka T (2001). Simultaneous validation of ten physical activity questionnaires in older men: a doubly labeled water study. J Am Geriatr Soc.

[19] Haskell WL, Lee I-M, Pate RR, Powell KE, Blair SN, Franklin BA (2007). Physical activity and public health: updated recommendation for adults from the American College of Sports Medicine and the American Heart Association. Med Sci Sports Exerc.

[20] Luben R, Hayat S, Wareham N, Khaw KT (2016). Predicting admissions and time spent in hospital over a decade in a population-based record linkage study: the EPIC-Norfolk cohort. BMJ Open.

[21] Ewald BD, Oldmeadow C, Attia JR (2017). Daily step count and the need for hospital care in subsequent years in a community-based sample of older Australians. Med J Aust.

[22] Simmonds B, Fox K, Davis M, Ku P-W, Gray S, Hillsdon M, et al. Objectively assessed physical activity and subsequent health service use of UK adults aged 70 and over: a four to five year follow up study. PLoS One. 2014;9. 10.1371/journal.pone.0097676. 10.1371/journal.pone.0097676 PMC4035293 24866573

[23] Ku P-W, Steptoe A, Chen Y-H, Chen L-J, Lin C-H (2017). Prospective association between late-life physical activity and hospital care utilisation: a 7-year nationwide follow-up study. Age Ageing.

[24] Syddall HE, Westbury LD, Simmonds SJ, Robinson S, Cooper C, Sayer AA. Understanding poor health behaviours as predictors of different types of hospital admission in older people: findings from the Hertfordshire Cohort Study. J Epidemiol Community Health. 2015. 10.1136/jech-2015-206425. 10.1136/jech-2015-206425 PMC4783823 26481495

[25] Day N, Oakes S, Luben R, Khaw KT, Bingham S, Welch A (1999). EPIC-Norfolk: study design and characteristics of the cohort. European Prospective Investigation of Cancer. Br J Cancer.

[26] Wareham NJ, Jakes RW, Rennie KL, Schuit J, Mitchell J, Hennings S (2003). Validity and repeatability of a simple index derived from the short physical activity questionnaire used in the European prospective investigation into Cancer and nutrition (EPIC) study. Public Health Nutr.

[27] Khaw K-T, Jakes R, Bingham S, Welch A, Luben R, Day N (2006). Work and leisure time physical activity assessed using a simple, pragmatic, validated questionnaire and incident cardiovascular disease and all-cause mortality in men and women: the European prospective investigation into Cancer in Norfolk prospective population study. Int J Epidemiol.

[28] Bland R (1979). Measuring “social class”: a discussion of the registrar-General’s classification. Sociology..

[29] McFadden E, Luben R, Khaw K-T (2009). Different measures of social class in women and mortality. Eur J Epidemiol.

[30] Hayat SA, Luben R, Keevil VL, Moore S, Dalzell N, Bhaniani A (2014). Cohort profile: a prospective cohort study of objective physical and cognitive capability and visual health in an ageing population of men and women in Norfolk (EPIC-Norfolk 3). Int J Epidemiol.

[31] Boyle S (2011). United Kingdom (England): health system review. Health Syst Transit.

[32] NHS England. Reference costs | NHS improvement. 2017. https://improvement.nhs.uk/resources/reference-costs/. Accessed 3 Jul 2019.

[33] NHS England. Statistics » bed availability and occupancy data – overnight. 2017. https://www.england.nhs.uk/statistics/statistical-work-areas/bed-availability-and-occupancy/bed-data-overnight/. Accessed 3 Jul 2019.

[34] OECD. OECD Health Statistics 2018 - OECD. 2018. http://www.oecd.org/els/health-systems/health-data.htm. Accessed 18 Apr 2019.

[35] Naveed S, David P (2017). Reverse causality in cardiovascular epidemiological research. Circulation..

[36] Sari N (2011). Exercise, physical activity and healthcare utilization: a review of literature for older adults. Maturitas..

[37] Biswas A, Oh PI, Faulkner GE, Bajaj RR, Silver MA, Mitchell MS (2015). Sedentary time and its association with risk for disease incidence, mortality, and hospitalization in adults: a systematic review and meta-analysis. Ann Intern Med.

[38] Tran B, Falster MO, Douglas K, Blyth F, Jorm LR (2014). Health Behaviours and potentially preventable hospitalisation: a prospective study of older Australian adults. PLoS One.

[39] Bijnen FCH, Feskens EJM, Caspersen CJ, Mosterd WL, Kromhout D (1998). Age, period, and cohort effects on physical activity among elderly men during 10 years of follow-up: the Zutphen elderly study. J Gerontol A Biol Sci Med Sci.

[40] Ahad W, Nishma M, Melanie N, Paul K, Charlie F, Premila W, et al. Quantifying the Association Between Physical Activity and Cardiovascular Disease and Diabetes: A Systematic Review and Meta-Analysis. J Am Heart Assoc. 2016;5:e002495. 10.1161/JAHA.115.002495 PMC5079002 27628572

[41] Sari N (2014). Sports, exercise, and length of stay in hospitals: is there a differential effect for the chronically ill people?. Contemp Econ Policy.

[42] Ding D, Lawson KD, Kolbe-Alexander TL, Finkelstein EA, Katzmarzyk PT, van Mechelen W (2016). The economic burden of physical inactivity: a global analysis of major non-communicable diseases. Lancet.

[43] Wang F, McDonald T, Reffitt B, Edington DW (2005). BMI, physical activity, and health care utilization/costs among Medicare retirees. Obes Res.

[44] Sari N (2010). A short walk a Day shortens the hospital stay: physical activity and the demand for hospital services for older adults. Can J Public Health.

[45] Li C-L, Chu S-J, Sheu J-T, Huang LY-G (2011). Impact of physical activity on hospitalization in older adults: a nationwide cohort from Taiwan. Arch Gerontol Geriatr.

[46] Morgan MDL (2003). Preventing hospital admissions for COPD: role of physical activity. Thorax..

