# Supplementary material for: Usual physical activity and subsequent hospital usage over 20 years in a general population: the EPIC-Norfolk cohort
Source: BMC Geriatr. 2020 May 6;20:165. doi: 10.1186/s12877-020-01573-0 (PMC7204050; doi:10.1186/s12877-020-01573-0)

Supplementary files

Filename: Supplementary_Tables_BMCGeriatrics_20200430.pdf

Supplementary Table S1 | Multivariable logistic regression of risk factors for any hospital admissions, ≥7 hospital admissions and >20 days of hospital stay from 1999 to 2019 in 25 014 men and women

Supplementary Table S2 | Adjusted mean hospital days by physical activity category for two periods, mean difference in days and cumulative cost, 1999–2009 using baseline physical activity and 2009–2019 using physical activity at TP2

Supplementary Figure | Flow diagram showing numbers of participants at various stages in the EPIC-Norfolk study including invitations, consents, attendance at health examinations and questionnaire completion.

Supplementary Table S1 | Multivariable logistic regression of risk factors for any hospital admissions, ≥7 hospital admissions and >20 days of hospital stay from 1999 to 2019 in 25 014 men and women

	All subjects OR (95% CI)	p value	Men OR (95% CI)	p value	Women OR (95% CI)	p value
**Outcome of any hospital admissions**						
Male sex	1.13 (1.03–1.23)	0.007				
Age per 10 years	2.07 (1.96–2.19)	< 0.001	2.15 (1.98–2.33)	< 0.001	2.01 (1.87–2.17)	< 0.001
Inactive	0.91 (0.82–1.01)	0.073	0.91 (0.78–1.07)	0.243	0.91 (0.80–1.05)	0.187
Manual social class	1.15 (1.05–1.26)	0.003	1.19 (1.03–1.38)	0.016	1.12 (0.99–1.27)	0.077
Lower education level	1.42 (1.27–1.58)	< 0.001	1.23 (1.04–1.47)	0.016	1.54 (1.35–1.77)	< 0.001
Current smoker	1.18 (1.03–1.35)	0.019	1.14 (0.93–1.40)	0.207	1.20 (1.01–1.45)	0.046
BMI>30 kg/m²	1.30 (1.14–1.49)	< 0.001	1.19 (0.97–1.47)	0.108	1.38 (1.16–1.64)	< 0.001
**Outcome of seven or more hospital admissions**						
Male sex	1.21 (1.15–1.28)	< 0.001				
Age per 10 years	1.45 (1.40–1.49)	< 0.001	1.45 (1.38–1.51)	< 0.001	1.45 (1.39–1.51)	< 0.001
Inactive	1.04 (0.98–1.10)	0.197	1.02 (0.93–1.11)	0.735	1.06 (0.98–1.15)	0.145
Manual social class	1.16 (1.09–1.23)	< 0.001	1.16 (1.06–1.26)	< 0.001	1.16 (1.07–1.25)	< 0.001
Lower education level	1.14 (1.07–1.21)	< 0.001	1.13 (1.03–1.23)	0.011	1.15 (1.06–1.24)	< 0.001
Current smoker	1.33 (1.23–1.45)	< 0.001	1.25 (1.11–1.41)	< 0.001	1.41 (1.26–1.58)	< 0.001
BMI>30 kg/m²	1.38 (1.28–1.48)	< 0.001	1.38 (1.23–1.54)	< 0.001	1.38 (1.25–1.52)	< 0.001
**Outcome of more than 20 hospital days**						
Male sex	1.14 (1.08–1.21)	< 0.001				
Age per 10 years	2.60 (2.52–2.70)	< 0.001	2.53 (2.41–2.66)	< 0.001	2.67 (2.54–2.80)	< 0.001
Inactive	1.12 (1.05–1.19)	< 0.001	1.08 (0.99–1.19)	0.084	1.14 (1.05–1.25)	0.002
Manual social class	1.18 (1.11–1.26)	< 0.001	1.20 (1.10–1.31)	< 0.001	1.17 (1.07–1.27)	< 0.001
Lower education level	1.16 (1.09–1.23)	< 0.001	1.18 (1.07–1.30)	< 0.001	1.14 (1.05–1.24)	0.002
Current smoker	1.55 (1.42–1.69)	< 0.001	1.53 (1.35–1.74)	< 0.001	1.56 (1.38–1.76)	< 0.001
BMI>30 kg/m²	1.58 (1.46–1.71)	< 0.001	1.54 (1.37–1.74)	< 0.001	1.61 (1.45–1.78)	< 0.001
**Outcome of 12 or more hospital admissions**						
Male sex	1.31 (1.22–1.41)	< 0.001				
Age per 10 years	1.26 (1.21–1.31)	< 0.001	1.31 (1.24–1.38)	< 0.001	1.22 (1.15–1.29)	< 0.001
Inactive	1.06 (0.98–1.15)	0.124	1.07 (0.96–1.20)	0.193	1.05 (0.94–1.17)	0.352
Manual social class	1.20 (1.12–1.29)	< 0.001	1.19 (1.07–1.32)	0.001	1.22 (1.10–1.35)	< 0.001
Lower education level	1.06 (0.98–1.15)	0.119	1.00 (0.90–1.12)	0.934	1.12 (1.01–1.25)	0.035
Current smoker	1.42 (1.28–1.57)	< 0.001	1.28 (1.10–1.48)	0.001	1.58 (1.37–1.82)	< 0.001
BMI>30 kg/m²	1.33 (1.21–1.45)	< 0.001	1.38 (1.21–1.58)	< 0.001	1.28 (1.13–1.45)	< 0.001
**Outcome of more than 50 hospital days**						
Male sex	1.02 (0.95–1.09)	0.647				
Age per 10 years	2.41 (2.32–2.51)	< 0.001	2.22 (2.10–2.36)	< 0.001	2.58 (2.44–2.74)	< 0.001
Inactive	1.12 (1.04–1.20)	0.002	1.03 (0.93–1.15)	0.558	1.19 (1.08–1.31)	< 0.001
Manual social class	1.17 (1.09–1.26)	< 0.001	1.21 (1.09–1.34)	< 0.001	1.14 (1.04–1.26)	0.007
Lower education level	1.15 (1.07–1.24)	< 0.001	1.16 (1.04–1.30)	0.006	1.14 (1.03–1.25)	0.011
Current smoker	1.56 (1.41–1.73)	< 0.001	1.51 (1.30–1.74)	< 0.001	1.61 (1.40–1.85)	< 0.001
BMI>30 kg/m²	1.48 (1.35–1.61)	< 0.001	1.46 (1.27–1.66)	< 0.001	1.50 (1.33–1.68)	< 0.001

Supplementary Table S2 | Adjusted † mean hospital days by physical activity category for two periods, mean difference in days and cumulative cost, 1999–2009 using baseline physical activity and 2009–2019 using physical activity at TP2

	Cohort survivors	Hospital Days inactive	Hospital Days any-activity	Difference in hospital days	Mean difference over period	Cumulative cost £
**1999–2009**						
1999	24 785	2.34	1.58	0.74	0.42	248
2000	24 528	1.78	1.46	0.34	0.42	495
2001	24 237	1.76	1.56	0.20	0.42	743
2002	23 916	2.16	1.68	0.48	0.42	991
2003	23 575	2.06	1.58	0.46	0.42	1239
2004	23 221	2.10	1.58	0.52	0.42	1486
2005	22 864	2.10	1.72	0.36	0.42	1734
2006	22 456	2.18	1.72	0.46	0.42	1982
2007	22 003	2.00	1.64	0.34	0.42	2230
2008	21 557	2.02	1.70	0.32	0.42	2477
**2009–2019**						
2009	9642	1.58	0.94	0.62	0.46	2746
2010	9533	1.52	1.16	0.38	0.46	3015
2011	9389	2.08	1.28	0.78	0.46	3283
2012	9222	2.16	1.76	0.42	0.46	3552
2013	9023	2.54	2.18	0.38	0.46	3821
2014	8859	2.24	1.96	0.28	0.46	4089
2015	8619	2.34	1.52	0.82	0.46	4358
2016	8362	2.12	1.46	0.66	0.46	4627
2017	8101	2.06	1.98	0.08	0.46	4895
2018	7948	1.68	1.52	0.18	0.46	5164

† Adjusted for age, sex, manual social class, lower education level, current cigarette smoker, body mass index > 30kg/m^2.

Supplementary Figure | Flow diagram showing numbers of participants at various stages in the EPIC-Norfolk study including invitations, consents, attendance at health examinations and questionnaire completion.

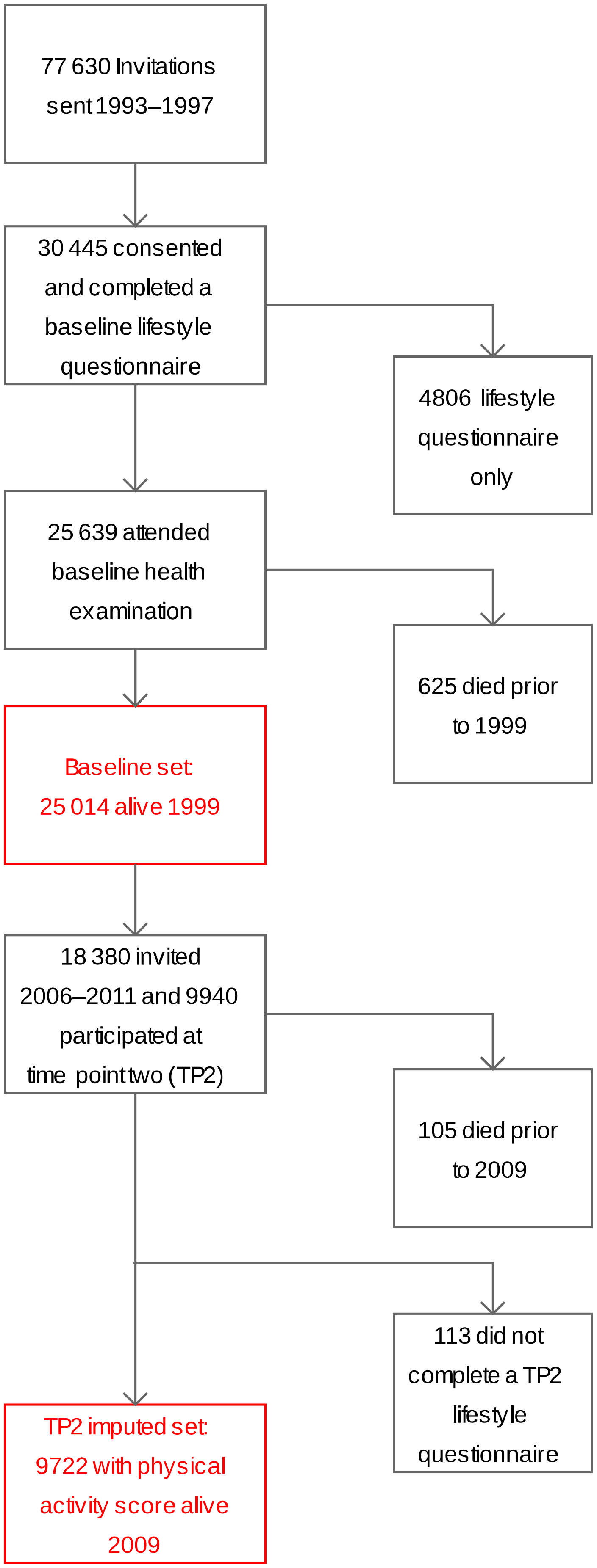

Supplement: Supplementary file 1 — Additional file 1: Table S1. Multivariable logistic regression of risk factors for any hospital admissions, ≥7 hospital admissions and > 20 days of hospital stay from 1999 to 2019 in 25,014 men and women. Table S2. Adjusted mean hospital days by physical activity category for two periods, mean difference in days and cumulative cost, 1999–2009 using baseline physical activity and 2009–2019 using physical activity at TP2. Figure S1. Flow diagram showing numbers of participants at various stages in the EPIC-Norfolk study including invitations, consents, attendance at health examinations and questionnaire completion. [file 12877_2020_1573_MOESM1_ESM.pdf]
